# Importance about use of high-throughput sequencing in pediatric: case report of a patient with Fanconi-Bickel syndrome

**DOI:** 10.1186/s12887-024-04641-1

**Published:** 2024-03-07

**Authors:** Hugo Hernán Abarca-Barriga, María Cristina Laso-Salazar, Diego Orihuela-Tacuri, Jenny Chirinos-Saire, Anahí Venero-Nuñez

**Affiliations:** 1 Instituto de Investigaciones de Ciencias Biomédicas, Universidad Ricardo Palma, Av. Benavides 5440, Santiago de Surco, Lima, Perú; 2 Servicio de Genética & Errores Innatos del Metabolismo, Instituto Nacional de Salud del Niño- Breña, Lima, Perú; 3 Equipo Funcional de Genética y Biología Molecular, Instituto Nacional de Enfermedades Neoplásicas, Lima, Perú; 4 Servicio de Endocrinología, Hospital de Emergencias-EsSalud, Lima, Perú

**Keywords:** *SLC2A2*, Hypophosphatemia, Glycogen storage disease, Renal tubular acidosis, Exome sequencing

## Abstract

**Background:**

Fanconi-Bickel syndrome is characterized by hepatorenal disease caused by anomalous glycogen storage. It occurs due to variants in the *SLC2A2* gene. We present a male patient of 2 years 7 months old, with failure to thrive, hepatomegaly, metabolic acidosis, hypophosphatemia, hypokalemia, hyperlactatemia.

**Results:**

Exome sequencing identified the homozygous pathogenic variant NM_000340.2(*SLC2A2*):c.1093 C > T (p.Arg365Ter), related with Fanconi-Bickel syndrome. He received treatment with bicarbonate, amlodipine, sodium citrate and citric acid solution, enalapril, alendronate and zolendronate, and nutritional management with uncooked cornstarch, resulting in an improvement of one standard deviation in weight and height.

**Conclusions:**

The importance of knowing the etiology in rare genetic disease is essential, not only to determine individual and familial recurrence risk, but also to establish the treatment and prognosis; in this sense, access to a new genomic technology in low- and middle-income countries is essential to shorten the diagnostic odyssey.

## Background

Fanconi-Bickel syndrome (OMIM #227810), an autosomal recessive inherited disorder, is characterized by a combination of liver and kidney disease caused by a defect in the glucose transporter GLUT2 (*SLC2A2* gene), which leads to an accumulation of glycogen, proximal renal tubular dysfunction, and failure to utilize glucose and galactose [1, 2].

The phenotype includes failure to thrive, a distended abdomen, hepatomegaly, fasting hypoglycemia, postprandial hyperglycemia, glucosuria, phosphaturia, aminoaciduria, polyuria, metabolic acidosis, osteoporosis, hypophosphatemia, rickets, and the presence of glycogen in liver or renal biopsy [1, 2]. In rare cases, hepatocellular carcinoma has been observed due to activation of the *Wnt* pathway [3]. However, patients with mild clinical manifestations have been reported, including those with only glucosuria [4].

The *SLC2A2* gene (OMIM *138160) contains 11 exons, and its GLUT2 protein is composed of 524 amino acids and is located in the cell membrane, expressed in hepatocytes, enterocytes, renal proximal tubules, pancreatic beta cells, neurons, and astrocytes [2]. Pathogenic variants of *SLC2A2* alter glucose entry and exit in hepatocytes and decrease insulin secretion due to increased sensitivity of beta cells in the postprandial phase [5].

Treatment involves preventing hypoglycemia and supplementing electrolytes [5]. However, classical nutritional management (frequent feeding plus overnight feeding) is suboptimal [6], therefore, continuously enteral nutritional uncooked cornstarch treatment at night and through the day has been proposed to improve growth [5].

This paper describes a patient with heterogeneous clinical features, raising different clinical diagnoses. However, exome sequencing determined a homozygous variant in the *SLC2A2* gene related to Fanconi-Bickel syndrome (FBS).

## Methods

Clinical information of the patient was obtained and evaluated at Instituto Nacional de Salud del Niño-Breña, Lima, Perú. DNA isolated from peripheral blood collected in EDTA using the *gSYNC DNA Extraction Kit* (Geneaid, Taiwan). DNA concentrations were determined using *Qubit Assays (Thermo Fisher Scientific*, USA). *The Ion AmpliSeq Exome RDY Kit (Thermo Fisher Scientific*, USA) was used for exome enrichment and library construction. The sample was ligated with an adapter and barcode using the *Ion Xpress kit*, and DNA purification was performed with *Agencourt Ampure XP beads (Beckman Coulter, Indianapolis*, IN, USA). The *Ion 540™ Kit-Chef* was used for templating and chip loading for sequencing. Exome sequencing was performed using a 400 bases read length and a total of 520 flows on an *Ion GeneStudio S5 sequencer (Life Technologies).* Reads were aligned to the hg19 reference genome, *Homo sapiens*, and variant calling, and annotation of single nucleotide variants (SNVs) were performed using the Varstation® platform. The parents of the patients did not undergo exome sequencing. The study was conducted in accordance with the tenets of the Declaration of Helsinki and approved by the Ethical Committee of Instituto Nacional de Salud del Niño Breña (INSN) (OEAIDE-6236-2022/UDISEÑO-055-2023); consent for publication was signed by the legal guardian (mother) trough informed consent.

## Results

**Fig. 1:**
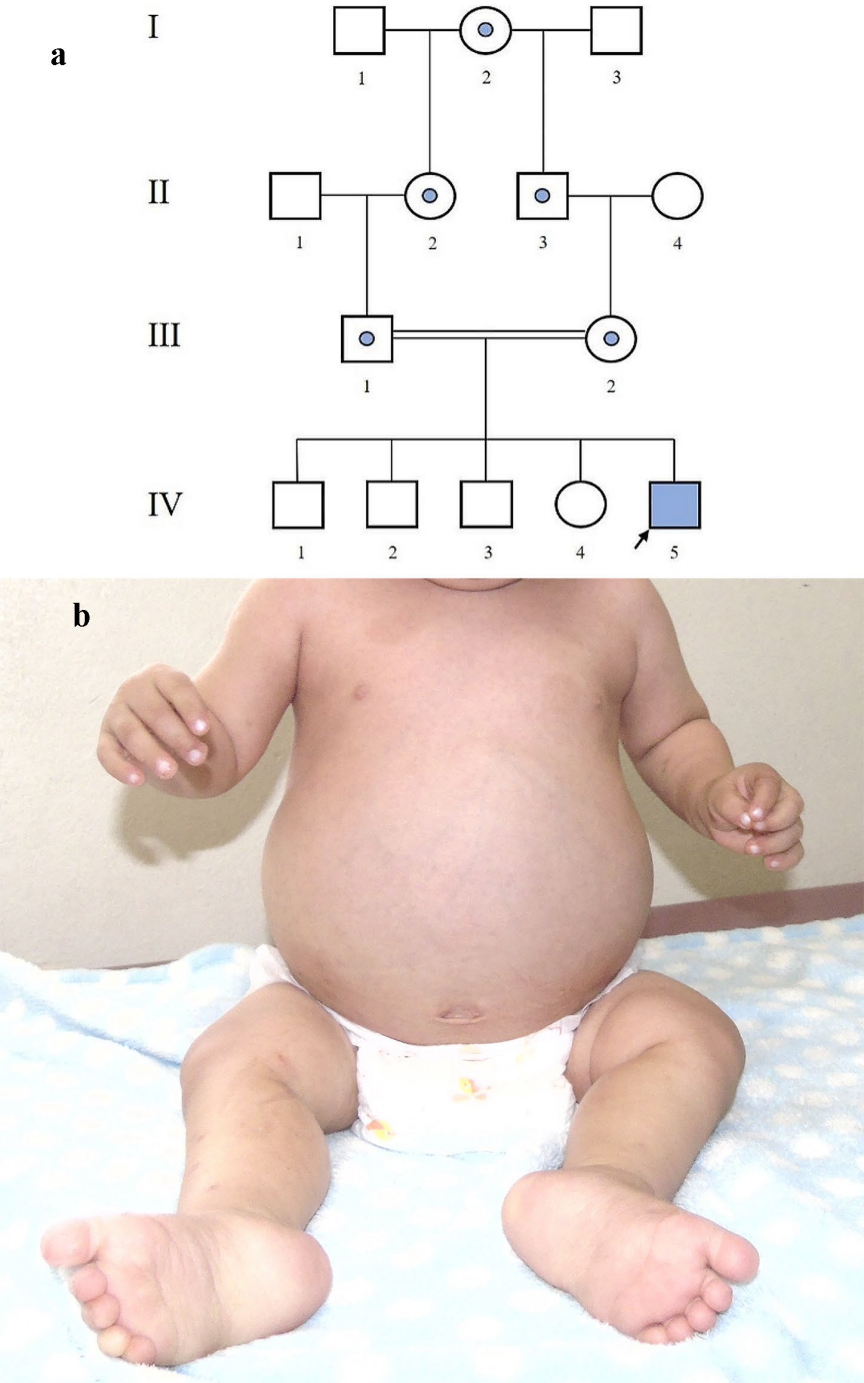
**a** Pedigree. Probably obligate carrier (patient had a homozygous variant): I-2; II-2; II-3; III-1; III-2. III-1: 50 years old. III-2: 37 years old. IV-1: 18 years old. IV-2: 16 years old. IV-3: 12 years old. IV-4: 5 years old. IV-5: 2 years old, Fanconi Bickel syndrome. **b** Photograph of the patient. Note the frontal bossing and increased abdominal volume

We present a 2 year and 7-month-old male patient, born and raised in Pucallpa (Peru), from the fifth pregnancy of consanguineous parents (Fig. 1A). He was born by vaginal delivery, full term, with a birth weight of 3620 g, height of 49 cm, and head circumference of 34 cm (normal percentiles), and with an Apgar score of 8–9. Regarding psychomotor development, he achieved head control at one month of age, sat unsupported at seven months, walked with support at one year and six months, spoke his first words at one year and five months, said two words at two years and nine months, and exhibited social smiling at one year.

He was evaluated at one year and eight months of age for poor weight and height gain, chronic diarrhea, fever, and increased abdominal volume. He was hospitalized three additional times (at 1 year and 10 months, 2 years and 2 months, and 2 years and 7 months) for food-like vomit, diarrhea, metabolic acidosis, hypokalemia and hyperlactatemia, hypoactivity, and fever.

**Fig. 2:**
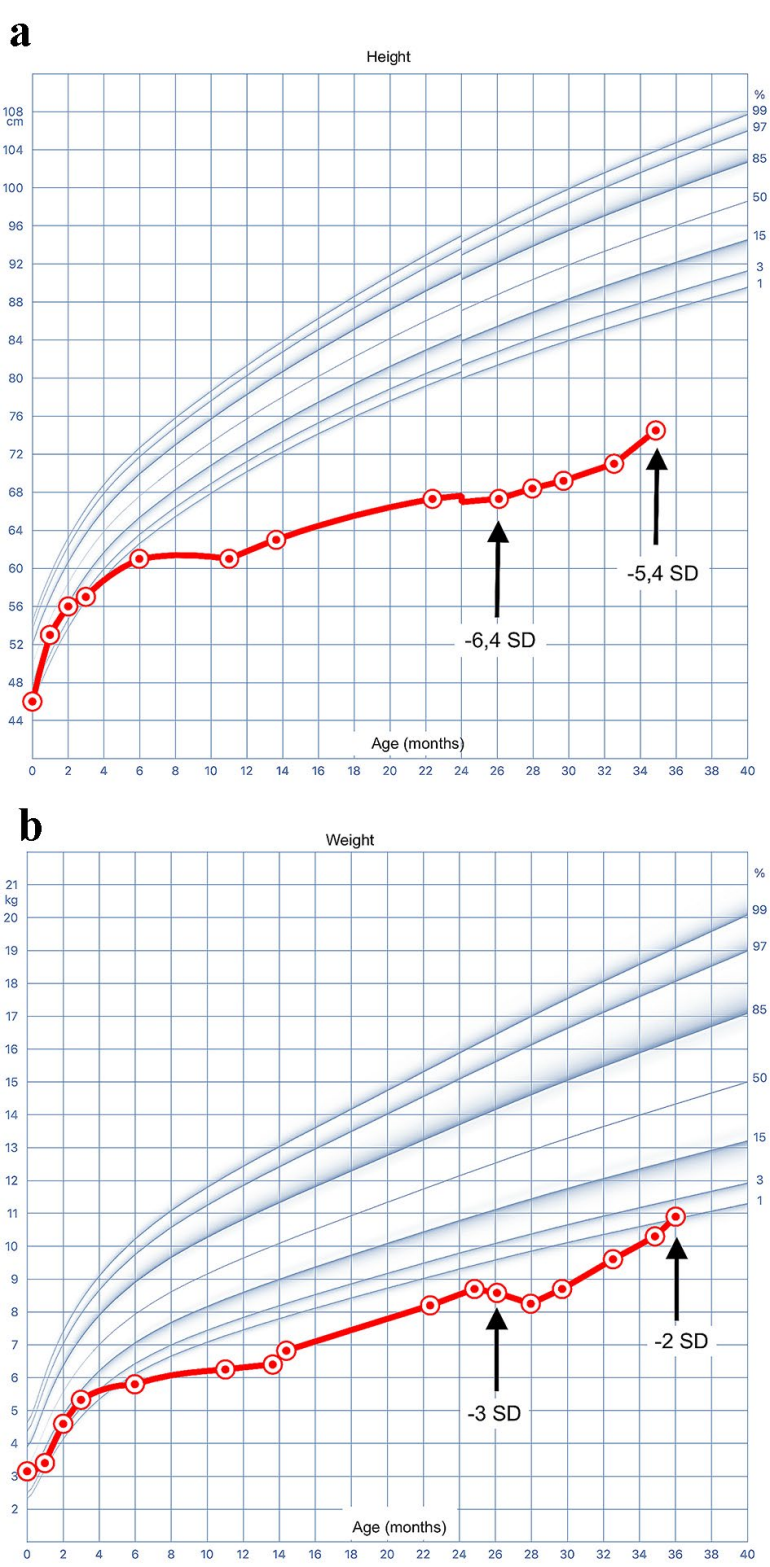
**a** Height/age growth curve in months. Low height for age is evident, however, an improvement in the curve is also observed from 26 months onward. **b** Weight/age growth curve in months. Low weight for age is evident, however, an improvement in the curve is also observed from 26 months onward, reaching the normal curves

During the physical examination, notable findings included frontal bossing, hepatomegaly, hypotonia, and pseudo-Madelung deformity (Fig. 1B). His weight and height were below the first percentile since six months of age (Fig. 2A and B), while his head circumference was within normal limits. Blood pressure was between 98–121/55–61 mmHg. X-Ray showed fraying and widening of metaphysis of femur (distal) and tibiae (proximal), compatible with rickets.

He showed hypoglycemia (20–64 mg/dl; NV = 70–99), hypo- and hypercalcemia (7,2–15,3 mg/dl; NV = 8,8–10,2), hypophosphatemia (1–3,9 mg/dl; NV = 4–7), hypercholesterolemia (166–334 ng/dL; NV = < 200), hypertriglyceridemia (291–1907 ng/dL NV = < 150), hyperphosphatasemia (1154–2937 U/L; NV = 0-645), hypokalemia during diarrhea and vomit (2,37 − 3,3 mmol/l; NV = 3,5–5,3), hyperlactatemia (2,1–8,0 mmol/l; NV = 0,4 − 1,8), and hypouricemia (1,1–2,7 mg/dl; NV = 2,5–5,5). Urine analysis showed a normal pH, density of 1031 g/ml, protein (1+) and glucose (3+), hyperproteinuria (68–169,7 mg/dl NV = < 25), hypocreatinuria (6–17 mg/dl; NV = 39–259), microalbuminuria (346–1830 mg/g Cr; NV = < 30), hyperglycosuria (290 mg/dl; NV = 0–15 mg/dl), and hypercalciuria (0,3 mg/dl; NV = 10,6-316). Additionally, he had thrombocytosis (465-778 × 10^3^/µl; NV = 150-450 × 10^3^). Venous gas showed pH 7,261-7,5 (NV = 7,38 − 7,46), mmHg, HCO^3^ 7,5–28,8 mmHg, and base excess − 15,8 to + 5,3. Urine analysis showed a normal pH, and normal HCO^3^ values (Table 1). These analyses led to the diagnosis of renal tubular acidosis. Total abdominal ultrasound showed hepatomegaly (LHD 131,6 mm), there was no evidence of fibrosis or nephromegaly.

**Table 1:** Summary of analysis at initial and last evaluation

Laboratory	Before UCS^a^	After UCS^b^	NV
Gamma-glutamil transpetidase	140	**322**	3–22 U/l
Aspartate transaminase	1454	136	0–47 mg/dl
Alanine transaminase	288	164	0–49 U/l
Triglicerides	592	**843**	< 150 mg/dl
Cholesterol	166	**268**	< 200 mg/dl
Phosphorus	1,7	3,9	4–7 mg/dl
Alkaline phosphatase	1054	**1584**	0-645 U/l
Lactic deshydrogenase	632	407	230–460 U/l
pH	7,261	7,305	7,38 − 7,46
PCO2	35,1	24,1	32–46 mmHg
pO2	44,4	57,9	74–108 mmHg
HCO3	15,2	**11,6**	
Base excess	-10,8	-13,3	
Calcium	10	10,4	8,8–10,2 mg/dl
Creatinine	0,28	0,35	0,30 − 0,70 mg/dl
Uric acid	1,3	2,5	2,5–5,5 mg/dl
Glucose	29	64	70–99 mg/dl
Urine pH	7,4^c^	8^c^	
Urine urea	182	--	
Urine calcium	3	3	mg/dl
Urine uric acid	21	**89**	mg/dl
Urine phosphorus	6	**26**	mg/dl
Microalbuminuria	346	**1830**	< 30 mg/g
Proteinuria	95	**315**	< 25 mg/dl
Urine creatine	9	29	39–259 mg/dl

Some laboratory data, particularly related to kidney function, did not show improvement (highlighted in bold fonts)

*UCS* Uncooked cornstarch, *NV* Normal values

^a^Initial evaluation

^b^Last control

^c^HCO^3^ plasma with normal values

A liver biopsy with a fine needle showed partially distorted hepatic architecture due to the presence of few fibrous enlargements of the portal space, inflammatory infiltration of lymphocytes and polymorphonuclear cells that do not exceed the limiting plate, large and ballooned hepatocytes with mosaic pattern and mild pericellular fibrosis. The periodic acid-Schiff with diastase highlights eosinophilic deposit within hepatocytes, which are correlated with deposit of glycogen. Therefore, considering these clinical and laboratory findings we suspected the diagnosis of FBS.

At age 1 year and 8 months, the genetic study results were obtained through genomic DNA. A total of 131 477 variants annotated in 18 179 genes were obtained, excluding variants that were likely benign or benign. To identify variants associated with the patient’s phenotype, the terms “Failure to thrive” (HPO: 0001508) and “hepatomegaly” (HPO: 0002240) were used, considering a population allele frequency threshold of 1%. Furthermore, we manually searched for variants in *SLC2A2*, because of hypophosphatemics rickets and glycogen storage in liver. Due to the parental consanguinity history, we prioritized the analysis of homozygous variants. A variant allele frequency (VAF) greater than 0,9 was used to select potential candidate genes. The identified variant was NM_000340.2(*SLC2A2*):c.1093 C > T (p.Arg365Ter), located in exon 9 at chromosome 3:170,716,931–170,716,931 [hg19], identified in ClinVar ® with ID: 16,092. Additionally, the analysis did not identify any other variants in compound heterozygosity or homozygosity related to autosomal recessive conditions, nor revealed heterozygous variants associated to autosomal dominant diseases that could be correlated with the described phenotype, secondary findings, or carrier status.

According to the American College of Medical Genetics and Genomics (ACMG) guidelines, the variant is classified as likely pathogenic. This alteration is a nonsense variant, generating a Stop codon at position 365 of the protein, predicted to undergo non-mediated decay. Loss of function in this gene is a known mechanism of disease. Additionally, exon 9, where the alteration is located, is present in biologically relevant transcripts, justifying the use of the PVS1 criterion. Although the variant has a very low reported frequency in gnomAD, meeting the PM2 criterion, according to Clingen recommendations, the PM2 criterion should be used as support. Clinvar ® classifies the variant as pathogenic, but considering the evidence described in the literature and the latest classification criteria, we classified the variant as likely pathogenic.

Currently, the patient receives maintenance treatment with bicarbonate (1 g every 8 h), oral citrate (Shohl’s) solution (1 ml = 1mEq; 10 ml every 8 h). Initially managed high blood pressure (because of chronic kidney failure) with amlodipine (1,25 mg per day), later switched to hydrochlorothiazide (12,5 mg per day). Additionally, for proteinuria the patient was prescribed enalapril (2,5 mg every 12 h), but did not show improvement. To address osteoporosis, the patient was taking alendronate (17,5 mg every week). However, due to availability in our institution, the treatment was subsequently switched to zoledronic acid (0,55 mg every 6 months), and nutritional maintenance treatment with uncooked cornstarch, which was initiated at 2 years and 3 months (8 g every 4 h; 1 g/kg). The patient did not experience any symptoms of hypoglycemia since the age of 2 years old. After UCS, symptoms of diarrhea were alleviated. In the last arterial blood gas analysis, the patients’ values were pH = 7,310 and HCO3^−^=12,4 mmHg. Anthropometry percentiles remained stable since the initiation of treatment. However, the patient continued to have elevated values of hepatic enzymes, with gamma-glutamyl transferase at 503 U/L (NV = 3–22), alanine transaminase at 2364 UL (NV = 0–39), and aspartate transaminase at 5362 (NV = 0–47).

## Discussion

The diagnostic odyssey in rare genetic diseases can take a patient up to 5–6 years to be accurately diagnosed [7], which can be even longer in low- and middle-income countries with limited technological resources, such as Peru [8]. The exome sequencing identified a homozygous nonsense variant in the *SLC2A2* gene, which has been described as pathogenic [9] based on three previously reported patients in homozygosity and four in compound heterozygosity [10]. In this context, while homozygous variants are more likely, we also searched for compound heterozygous variants related to the clinical diagnosis. The patients reported in the USA and Turkey do not provide detailed descriptions of the phenotype. In these cases, the majority are compound heterozygotes, and less frequently, homozygotes; however, they refer to a clinical and biochemical phenotype consistent with Fanconi-Bickel syndrome [6, 10]. One of the compound heterozygous patient (Japan) presented with glucosuria, aminoaciduria, disorder of phosphate reabsorption, accumulation of glycogen in the liver and negative enzymes related to glycogen deposition [11]. The differential diagnosis includes glycogen storage disease (GSD) type1, severe GSD3, and fructose 1,6 bisphosphonate deficiency (Table 2).

**Table 2:** Differential diagnosis of Fanconi-Bickel disease

Disorder	Gene	Location	Common phenotype with FBS	Differences with FBS	Inheritance
Glycogen storage disase type 1	*G6PC*	17q21.3	Hepatomegaly, fasting hypoglycemia, elevated transaminases; facies doll-like; intermittent diarrhea.	Hypertension often detected in the second life; hepatic adenomas, reduced von Willebrand factor	AR
Glycogen storage disase type 3	*AGL*	1p21.2	Hepatomegaly, fasting hypoglicemia, elevated transminases, osteoporosis.	Midface hypoplasia; cardiomyopathy; muscle weakness; normal blood lactate; type 2 diabetes mellitus	AR
Fructose 1,6 bisphosphonate	*FBP1*	9q22.32	Hepatomegaly, fasting hypoglycemia, elevated transaminases; hypotonia.	Onset in newborns tachycardia, sorbitol and glycerol intolerance; episodic of acute crisis (pulmonary or neurologic); pseudohypertrigliceridemia; lactic acidosis triggered by fasting or febrile infection	AR

Modified by the authors from www.omim.org and GeneReviews®

*G6PC* Glucose-6-phosphatase, *FBP1* Fructose-1,6-bisphosphatase deficiency, *AGL* Amylo-1,6-glucosidase

The clinical diagnosis of the patient was made based on the presence of hepatomegaly, hypoglycemia, glucosuria, hypophosphatemia, hyperphosphatasia, hypouricemia, rickets, pseudo-Madelung deformity, and renal tubular acidosis. However, the presence of aminoaciduria could not be established due to the absence of the test in the local setting (e.g., tandem mass spectrometry-MS/MS-) [12]. This inadequate excretion of amino acids would have facilitated the clinical-biochemical diagnosis. Nevertheless, molecular confirmation through the second step using Sanger sequencing of the *SLCA2* gene has been another obstacle due to the absence of this test in Peru, which highlights the technological gap within the country [8]. Considering the limited access to genetic studies in the country, it was not possible to confirm the presence of the variant in the patient’s first-degree relatives. Newborn screening in Peru is currently limited to four entities, employing outdated technologies. Tests such as the measurement of galactose levels (used as a screening method for FBS) and other tests like MS/MS or high-performance liquid chromatography (to detect fatty oxidation disorders, certain organic acidurias and amino acidopathies) are not available due to political decisions. While these technologies, specifically exome sequencing, are not currently employed for neonatal screening in high-income countries yet, they are useful for the diagnosis of other inborn errors of metabolism like FBS.

Nevertheless, at INSN in Peru, alternative, and likely more efficient technologies are employed. These technologies are capable of detecting a broader range of genetic diseases, including inborn errors of metabolism, as well as patients exhibiting phenotypes characterized by neurodevelopmental disorders or congenital anomalies, considering their prevalence. Thus, new techniques of massive sequencing allow the evaluation of from a dozen genes up to the whole genome of the patient; increasing the efficiency in the etiological diagnosis [13], and improving the cost-effectiveness relationship compared to other tests [14]. Exome sequencing allowed us to accurately identify a homozygous variant. However, in numerous cases, a complementarity relationship between biochemical and massive sequencing tests has been observed, mainly in instances where variants of uncertain significance are found or that the phenotype is unspecific [15].

The observed elevation in blood lactate could be attributed to an increased glucose load trough anaerobic metabolism [16]. Additionally, low uric acid is part of the phenotype in FBS patients due to dysfunction of proximal tubular, resulting in hyperuricosuria [1, 2].

Some clinical characteristics, such as speech delay, are likely associated with the presence of chronic hypoglycemia.

Furthermore, it is essential to highlight that, since the beginning of the dietary treatment with uncooked cornstarch, the patient showed an improvement of 1SD in weight and height (Fig. 2A and B). Therefore, its use is essential not only to prevent nocturnal hypoglycemia but also to improve the height and weight of patients.

## Conclusion

It should be mentioned that, despite the challenges associated with identifying the etiology of rare genetic diseases, describing it has a significant importance. This is because it establishes the risk of familial and individual recurrence, determining prognosis, and guiding the application of the best therapeutic measures. As seen in the case of the patient, access a comprehensive and multidisciplinary management has positively impacted his growth. In this sense, implementing genomic technologies in our environment will gradually shorten the diagnostic odyssey, benefiting more patients to establish early diagnosis and management, in order to improve their health and quality of life, as well as providing proper family counseling.

## Data Availability

Not applicable for that section.
